# Prevalence of *Cryptosporidium*-like infection in one-humped camels (*Camelus dromedarius*) of northwestern Iran

**DOI:** 10.1051/parasite/2012191071

**Published:** 2012-02-15

**Authors:** M. Yakhchali, T. Moradi

**Affiliations:** 1 Department of Pathobiology, Parasitology Division, Faculty of Veterinary Medicine, Nazlu campus, Urmia University Urmia city West Azerbaijan province PO BOX 57153-1177 Iran; 2 Laboratory technitioner in Miandoab Iran

**Keywords:** *Cryptosporidium*, protozoan, prevalence, livestock, camel, Iran, *Cryptosporidium*, protozoaire, prévalence, bétail, dromadaire, Iran

## Abstract

*Cryptosporidium* is a ubiquitous enteropathogen protozoan infection affecting livestock worldwide. The present study was carried out to determine the prevalence of *Cryptosporidium* infection in different age groups of dromedary camels in northwestern Iran from November 2009 to July 2010. A total number of 170 fecal samples were collected and examined using modified Ziehl-Neelsen (MZN) staining under light microscope. Examination of stained fecal smears revealed that 17 camels (10%) were positive for *Cryptosporidium-*like. The prevalence of *Cryptosporidium*-like was significantly higher in camel calves (< 1 years old) (20%) than other age groups, in which the diarrhoeic calves had the prevalence of 16%. In adult camels the prevalence was 6.5%. There was no significant difference in the prevalence of *Cryptosporidium*-like between male and female camels. It is concluded that *Cryptosporidium* infection is a problem in camel husbandry and could be of public health concern in the region.

## Abstract (fr)

Les *Cryptosporidium* sont des protozoaires entéropathogène qui infectent le bétail partout dans le monde. L’étude avait pour objectif de déterminer la prévalence de l’infection à *Cryptosporidium* chez des dromadaires de différents groupes d’âge au nord-ouest de l’Iran, de novembre 2009 à juillet 2010. Au total, 170 échantillons de fèces ont été collectés et examinés en microscopie optique après coloration de Ziehl-Neelsen modifiée (MZN). L’examen a montré que 17 dromadaires (10 %) étaient porteurs de *Cryptosporidium*. Chez les jeunes de moins d’un an, la prévalence (20 %) était significativement plus élevée que celle des autres groupes d’âge. La prévalence la plus élevée (16 %) a été observée chez les jeunes atteints de diarrhée, et la moins élevée (6,5 %) chez les adultes non diarrhéiques. Il n’a pas été observé de différence significative de prévalence entre les males et les femelles. En conclusion, l’infection à *Cryptosporidium* chez les dromadaires pourrait poser un problème de santé vétérinaire dans la région.

*Cryptosporidium* species belong to the Apicomplexa phylum of parasites and have been detected in a wide range of hosts, including 155 mammalian species from many geographical regions of the world (Fayer & Ungar, 1986; Fayer, 2004). Infected human cases with *Cryptosporidium* spp. had a history of being in close contact with different species of domestic animals like camels. Some of the zoonotic *Cryptosporidium* species (*C. parvum*, *C. meleagridis*, *C. canis*) usually causes self-limiting diarrhoea in human and animals and could be a great public health concern worldwide (Minas *et al.*, 1994; de Graaf et al., 1999; Castro-Hermida *et al.*, 2002; Causapé *et al.*, 2002; Graczyk *et al.*, 2003; Joachim, 2004; Caccio, 2005; Wang *et al.*, 2008) and Iran (Mirzaei, 2007; Nahrevanian *et al.*, 2007; Yakhchali & Gholami, 2008; Razavi *et al.*, 2009).

The Camelidae family under the suborder Tylopoda is broken down into the *Lama* genus (New World Camelids) and the *Camelus* genus (Old World Camelids) including: *Camelus bactriamus* (Linnaeus, 1758; Asiatic or two-humped camel) otherwise known as the Bactrian camel and *Camelus dromedarius* (Linnaeus, 1758; Arabian or one-humped camel) or simply the camel (Wernery & Kdaden 2002; Yakhchali & Chraghi, 2007). There are about 20 million camels in North and East Africa countries, and Middle and Far East countries (Yakhchali & Athari, 2010). In Iran, one-humped camels are important multipurpose animal. The camels are the most suitable species of domestic mammals to be used under extremely arid conditions due to physiological attributes (Oryan *et al.*, 2008).

So far, 20 valid species of *Cryptosporidium* have been reported which 12 of them infecting mammals (Fayer, 2010) and over 40 genotypes with no species names described (Xiao *et al.*, 2004). Livestock cryptosporidiosis may have an important economic impact because of high morbidity and sometimes high mortality rates among animals (Sunnotel *et al.*, 2006). Despite its wide distribution and obvious relevance to animal health, *Cryptosporidium* prevalence in camels of northwestern Iran has not been yet reported and this is the first extensive and quantitative investigation on the dromedary camels’ cryptosporidiosis. For this purpose, the present study was carried out to determine the prelevance of *Cryptosporidium* infection in one-humped camels of Iranian farms.

## Materials and Methods

### Field Study Area

**Fig. 1.**
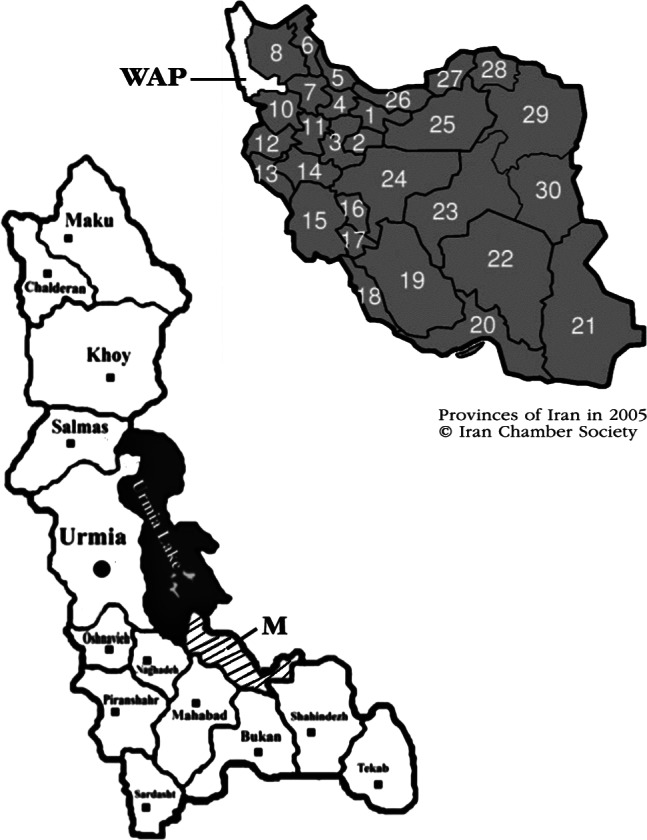
Geographical location of farms where animals sampled in northwestern Iran (M: Miandoab suburban; WAP: West Azerbaijan province).

The Miandoab region is located in the southeast of West Azerbaijan province between latitude 36° 57’ N and longitude 46° 06’ E (Fig. 1). An average population of more than 200,000 dromedary camels is distributed over vast camel-raising areas in the arid and semiarid deserts of the country (Razavi *et al.*, 2009). Approximately 0.3% of this population exist in northwestern Iran, especially in Miandoab suburban (Yakhchali & Athari, 2010).

### Sampling Procedure

The study was undertaken in the Miandoab suburban from November 2009 to July 2010. In the course of the study, a total number of 170 camels (85 males and 85 females) were randomly selected from camel farms using table of random numbers. Sample size was calculated according to Thrusfield (1997). The examined camels were crossbred and indigenous which reared traditionally. For each animal, age and sex were recorded. The age was determined on the basis of eruption of permanent incisor teeth (Smallwood, 1992). The animals were divided into four groups, namely young camel calves (less than one year old), immature (two-three years old), adult (four-five years old) and old (more than five years old). The animals subjected to a clinical examination including general body condition, heart and respiratory rates and signs of diarrhoea.

In each farm, fecal sample was collected directly from the rectum using sterile plastic gloves. The samples were transported to the laboratory in a cool box and then stored for a maximum of 24 h before analysis. The feces were classified according to their consistency as diarrhoeic (D) and non-diarrhoeic (ND).

### Sample Processing

One hundred and seventy stool samples were examined in the study. Each camel was numbered and subjected to a clinical examination. The collected fecal samples of each animal were examined by direct smear techniques. To determine oocysts shedding, the negative faecal samples by direct smear examination were concentrated by centrifugal sedimentation (2,500 rpm for 2 min) and Clayton-Lane flotation techniques using standard Sheather solution (sg 1.12) (Soulsby, 1982; Hendrix, 1998). The presence of *Cryptosporidium*-like oocysts in all samples was confirmed by modified Ziehl-Neelsen (MZN) staining (Henricksen & Polenz, 1981). The diameter of 100 *Cryptosporidium-*like oocysts of each infected camel was measured at 1,000 × magnification. Each positive sample was considered when at least one oocyst with the correct morphologic characters was observed (*Cryptosporidium*-like oocysts were 4-6 μm and spherical containing a residuum, sporozoites and usually within a clear halo, against a blue background) (Soulsby, 1982; Baxby *et al.*, 1984).

### Statistical Analysis

Statistical evaluation was undertaken to compare the prevalence among different age groups and gender with confidence interval of 95% using non-parametric Chi-square and *t* tests (SPSS for Windows). Probability value of < 0.05 was regarded statistically significant.

## Results

**Fig. 2.**
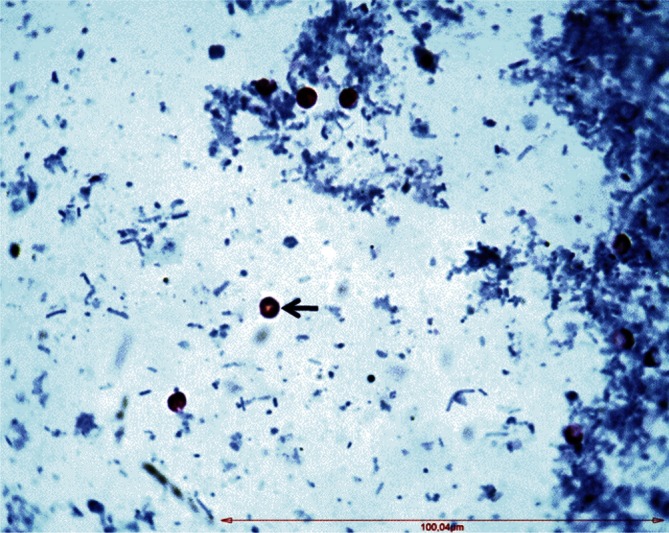
*Cryptosporidium*-like oocysts stained by modified Ziehl-Neelsen (MZN) method (arrow head, 1,000 ×).

The overall prevalence of *Cryptosporidium* species among the examined camels was 10% (17/170) (Table I). The parasite genus identification of the oocysts was confirmed by morphology. The oocysts were nearly spherical in shape and contained four sporozoites. The average size (± SD) of the oocysts was 5.70 (0.49) × 4.74 (0.3) μm (range 5.20-6.10 × 3.90-4.98 μm) with shape index (length/ width) of 1.19 (0.05, range 1.08-1.26). These morphological characters nearly fit with the description of *Cryptosporidium*-like oocysts (Fig. 2).

**Table I.** The prevalence of *Cryptosporidium* infection, oocysts shedding and faecal consistency, in different age groups of naturally infected camels in Miandoab suburb, Iran (n = 170).

					Fecal consistency (%)	
Age (year)	No. of examined animals	No. of infected animals	Prevalence (n/N) (%)	Oocyst shedding (%)	D	ND
< 1^a^	44	9	20.0	78	16	5
2–3	65	4	6.0	17	3	3
4–5	41	4	6.5	5	0	10
> 5	20	0	0.0	0	0	0
Total	170	17	10.0	100	5	5

a : x^2^ test (*p* < 0.05); D: diarrhoeic; n: number of infected animals; N: number of examined animals; ND: non-diarrhoeic.

Camel calves (< one year old) had the highest prevalence, with an overall average of 20% in the course of study. The prevalence was significantly higher (*p* < 0.05) in camel calves compared to the other age groups (Table I). Some of the infected animals showed wasting, diarrhoea and debility. Older camels with shedding oocysts in faeces did not show symptoms of cryptosporidiosis. The highest prevalence of *Cryptosporidium* species in D animals was 16% in camel calves. While in ND cases, it was 10% for adult camels (Table I). The oldest animal shedding *Cryptosporidium*-like oocysts was 4.5 years old. No old camels were infected with *Cryptosporidium* species at any time during the study.

The prevalence of infection in male and female examined camels indicated that five (6%) male and 12 (14%) female had *Cryptosporidium* infection. Infection rate was highest in female camels (33%) with less than one year old. There was no significant difference in the prevalence between male and female camels in all age groups (*p* > 0.05).

## Discussion

*Cryptosporidium* biology, distribution pattern, pathology and prevalence have been reported in many countries throughout the world. However, it is restricted to domestic ruminants (cattle, sheep and goats) and limited data is available in other herbivores including camels (Nouri, 2002; Razavi *et al.*, 2009). The results of present study revealed that *Cryptosporidium* infection occurred also in one-humped camels of northwestern Iran with low prevalence. The prevalence in naturally infected camels of Iran was reported in north-east (1.9%) (Borji *et al.*, 2009), south (37.9%) (Razavi *et al.*, 2009) and Qeshm Island in Persian golf (16.9%) (Nazifi *et al.*, 2010). Saleh & Mahran (2007), El Kelesh *et al.* (2009), Abdel-Wahab and Abdel-Maogood (2011) noted that camel *Cryptosporidium* infection rate in Egypt varied between 3.37-19.30%. These variations could be due to the difference in the environmental condition and hygienic measures.

In this study, age of examined camel calves had significant effect on prevalence compared to other age groups. According to Lorenzo *et al.* (1993), Scott *et al.* (1995) and Olson *et al.* (1997), young animals are much more susceptible to the infections than adult ones in other animals. These findings suggest that the age-related distribution of *Cryptosporidium* infection in this age group is not similar to that previously reported in camels (Soltane *et al.*, 2007; Borji *et al.*, 2009; Razavi *et al.*, 2009). It seems that the adult camels (three-four years old) with low prevalence, having normal formed of faeces (ND) and no clinical symptoms of cryptosporidiosis served as carriers for young camels.

The sex of examined camels had no significant effect on prevalence. With respect to this finding, the current study is in concordance with other researches (Chalmers *et al.*, 1997; Bull *et al.*, 1998; Razavi *et al.*, 2009). No infection in old camels (> five years old), even when camel calves were infected, suggests that immunity does develop in older animals (Fayer, 2004). It is therefore camels to be as healthy carriers and sources of *Cryptosporidium* infection for human beings and other animals. The infected animals can shed oocysts into the environment and remain as a source of infection to other animals and humans (Xiao *et al.*, 1993). Hence, some of the *Cryptosporidium* species (*C. parvum*, *C. meleagridis* and *C. canis*) are of zoonotic concern and could be of great public health concern in the region (Graczyk *et al.*, 2003).

Camel husbandry has been considered a sector of food supply for rural and sometimes urban people in this geographical region of Iran. Thus, their health status is important and epidemiological investigation on *Cryptosporidium* infections is useful to launch an all-round control programmed in this area. Therefore, further investigations will reveal more information about economic effects of this parasite and public health concern in the region.
